# Supplementary material for: WordCommentsAnalyzer: A windows software tool for qualitative research
Source: F1000Res. 2018 Sep 4;7:536. Originally published 2018 May 3. [Version 2] doi: 10.12688/f1000research.14819.2 (PMC6137411; doi:10.12688/f1000research.14819.2)
Supplement: Supplementary file 3 [file f1000research-7-17614-s0002.tgz › b2a79496-4ada-47c4-9682-a99ce867836d.zip!/Eight Iranian Health Journals/Journal of Health System Research _21.docx]

© Journal of Health System Research . This work is licensed under CC BY-NC 4.0 (https://creativecommons.org/licenses/by-nc/4.0/).

Volume & Issue: دوره 9، شماره 11؛ 1392

Title: مروری بر انواع مطالعات مروری در حوزه سلامت

http://hsr.mui.ac.ir/index.php/jhsr/article/view/1042

مطالعات مروری به نوعی از پژوهش اطلاق می‌گردند که جمعیت مورد مطالعه آن را، مقالات اصیل چاپ شده در یک حوزه خاص از دانش بشری تشکیل می‌دهند. هدف از نگارش مقاله مروری حاضر، معرفی انواع مطالعات مروری، همراه با شناخت نقاط قوت و ضعف هر یک از انواع این مطالعات در حوزه سلامت می‌باشد. در پایان سال 2012 میلادی موتور جستجوی پاب مد با بررسی واژگان “systematic reviews”  و  “meta-analyses”در عنوان مقاله و واژه “methodology” در عنوان یا خلاصه مقاله مورد جستجو قرار گرفت. سپس، با تغییر در روش جستجو و هم‌چنین با بهره‌گیری از منابع مورد استفاده در مقالات به دست آمده، دامنه جستجو تکمیل گردید. شیوه انجام مطالعات مروری در حوزه سلامت در طول زمان دستخوش تغییر و تحولات اساسی گشته و این قبیل مطالعات از نوع سنتی به منظم و سپس به فراتحلیل‌های به عمل آمده بر روی یافته‌های منتشر شده ارتقا یافته‌اند. فراتحلیل با بهره‌گیری از داده‌های تک‌تک شرکت‌کنندگان و تجزیه و تحلیل‌های آینده‌نگر برنامه‌ریزی شده مشترک، مطالعات جدیدتری هستند که به تازگی پیشنهاد شده‌اند. با شناخت صحیح نقاط قوت و ضعف و همچنین موارد کاربرد انواع گوناگون مطالعات مروری است که می‌توان از انجام این قبیل مطالعات، بیشترین بهره را در حوزه علوم سلامت به دست آورد.

Title: نقاط کور در برنامه اعتباربخشی

http://hsr.mui.ac.ir/index.php/jhsr/article/view/1065

اعتباربخشی یکی از ابزارهای ارزشیابی سیستم های بهداشتی درمانی به خصوص بیمارستان ها است که در ایران نیز در صدر اولویت های وزارت بهداشت، درمان و آموزش پزشکی قرار گرفته است اما سایر کشورهایی که برنامه اعتباربخشی را اجرا نمودند با چالش هایی رو به رو بوده اند. از آنجایی که برنامه اعتباربخشی در مراحل اولیه پیاده سازی در ایران است، تحقیق حاضر به صورت متفاوت، با بررسی سایر مطالعات به دنبال جنبه های منفی احتمالی در برنامه اعتباربخشی بوده تا بدین طریق چالش های موجود در اجرای این برنامه را که از دیده ها پنهان مانده استخراج کرده و راهکارهای پیشنهادی در جهت رفع آنان در ایران ارائه دهد. یافته های این مطالعه در پایان سال 91، به صورت مروری نقلی جمع آوری گردید و چالش های موجود در دسته های مجزا شامل؛ افزایش هزینه، عدم قطعیت در خصوص اثربخشی برنامه، تعارض بین تضمین کیفیت و بهبود کیفیت، استفاده از یک مجموعه واحد از استانداردها برای ارزیابی سازمان و برنامه ها در محیط های مختلف، ترویج بروکراسی اداری و دسته ای در ارتباط با کارکنان شامل؛ افزایش بارکاری آنان وکمبود نیروی انسانی، ایجاد استرس در کارکنان به دلیل سختی و زمان بر بودن اجرای آن قرار گرفته و بررسی شد. وجود این چالش ها در برنامه اعتباربخشی مانع از اجرای صحیح و رسیدن به هدف اعتباربخشی در سازمان ها می گردد. بنابراین این مطالعه به مسوولین در جایگاه سیاست گذاری پیشنهاد می دهد برای اجرای موفق این برنامه در ایران، تمام موارد شناسایی شده را از جوانب مختلف، مطالعه و در برنامه ریزی های خود جهت پیاده سازی برنامه در سطح کشور آن نکات را مدنظر قرار داده و با تشکیل جلسات و همایش های تخصصی در این زمینه برای یافتن راه حل های موثر اقدام لازم در جهت کاهش یا رفع اثرات آن انجام دهند.

Title: بررسی نقش اوقات فراغت مبتنی بر فعالیت های بدنی بر سرمایه اجتماعی(هنجارهای اجتماعی، مشارکت) اعضای هیئت علمی دانشگاه علوم پزشکی اصفهان

http://hsr.mui.ac.ir/index.php/jhsr/article/view/1218

مقدمه: تحقیقات متعددی نشان داده‌اند که ورزش و فعالیت بدنی با کاهش در استرس، اضطراب، افسردگی و بد خلقی و افزایش در خوش خلقی یا سلامت روانی و احساسات تجدید حیات در ارتباط است. کارکرد اجتماعی تربیت بدنی و ورزش در توسعه و تحکیم روابط اجتماعی، کارکرد زیر بنایی و بنیادی است که در کل ساختار جامعه و روابط موجود در بین اقشار و آحاد اجتماعی اثرات قابل توجهی بر جای می‌گذارد. هدف از انجام این پژوهش بررسی رابطه بین ورزش در اوقات فراغت و سرمایه اجتماعی اعضا هیأت علمی دانشگاه علوم پژشکی اصفهان بوده است.   روش‌ها: مطالعه از نوع توصیفی همبستکی بر روی اعضاء هیت علمی دانشگاه علوم پزشکی اصفهان انجام گرفت است. که گرد آوری داده ها از طریق دو پرسشنامه انجام گرفت. پرسشنامه سرمایه اجتماعی بر اساس مدل اسکات SCAT) )، و همچنین پرسشنامه اوقات فراغت محقق ساخته که روایی صوری و محتوای آنها مور تائید صاحبنظران قرار گرفته و نیز پایایی آن ها با استفاده از روش ضریب آلفای کرونباخ به ترتیب 92/. و 82/. بدست آمد. حجم نمونه با یک مطالعه مقدماتی و با استفاده از فرمول کوکران 150 نفر در نظر گرفته شد که به روش طبقه ای تصادفی با توجه به متغیر جنسیت و دانشکده انتخاب گردید. برای تحلیل داده ها از آزمون های T تک متغیره و آزمون صریب همبستگی پیرسون و رگرسیون چند گانه استفاده شد.   یافته‌ها: نتایج نشان داد که میانگین نمره اوقات فراغت افراد برابر با 42/3 و سرمایه اجتماعی افراد برابر 47/3 بوده است. همچنین تحلیل‌های رگرسیونی نشان داد، بین ترکیبی از مولفه‌های سرمایه اجتماعی (اعتماد و مشارکت) با میزان و نحوه گذراندن اوقات فراغت فیزیکی رابطه معنی‌داری وجود دارد. (602 R = ./ و P-value ≤ 0.05) و مقدار ضریب همبستگی چندگانه، یعنی ترکیبی از مولفه‌های سرمایه اجتماعی و اوقات فراغت فیزیکی 602/. و ضریب تعیین 362/. می‌باشد. این ضریب نشان می‌دهد که 2/36٪ از واریانس نمرات اوقات فراغت فیزیکی به وسیله ترکیبی از مولفه‌های سرمایه اجتماعی قابل توصیف، توضیح و تبیین می‌باشد.   نتیجه‌گیری: ورزش و فعالیت‌های بدنی در اوقات فراغت و شرکت فرد در فعالیت‌های گروهی و عضویت در آنها شرایطی را فراهم می‌سازد تا فرد با تعامل و برقراری ارتباط با سایر افراد و احساس تعلق به گروه، منافع و مصالح جمع را محترم بشمارد. این چنین فعالیت‌ها می‌تواند باعث افزایش سطح سرمایه اجتماعی و مولفه‌های آن نظیر، هنجارهای اجتماعی و مشارکت افراد گردد.

Title: بررسی آگاهی، خطر درک شده و قصد رفتاری دانشجویان دانشگاه علوم پزشکی اصفهان در خصوص هپاتیت B، سال 1391

http://hsr.mui.ac.ir/index.php/jhsr/article/view/1194

مقدمه: هپاتيت Bاز عمده‌ترين مشكلات سلامتی عصر حاضر و از علل افزایش خطر ابتلا به کارسینوم هپاتوسلولار و سیروز کبدی می‌باشد. دانشجویان علوم پزشکی به دلیل وضعیت شغلی آینده، جز گروه در معرض خطر و بیش از جمعیت عادی به بیماری مبتلا می‌شوند. مطالعه حاضر به بررسی میزان آگاهی، خطر درک شده و قصد رفتاری دانشجویان علوم پزشکی در خصوص هپاتیت B پرداخته است. روش‌‌ها: نوع مطالعه توصيفي – تحليلي و جمعیت مورد مطالعه دانشجویان علوم پزشکی اصفهان می‌باشند. نمونه‌گیری به روش تصادفی ساده و حجم نمونه بر اساس فرمول حداقل 385 نفر به دست آمد. ابزار جمع‌آوری اطلاعات، پرسشنامه و تجزیه و تحلیل داده‌ها با استفاده از نرم‌افزارSPSS  نسخه 18 و آزمون‌های آماری پیرسون، اسپیر من، تی‌مستقل و آنالیز واریانس یک‌طرفه بود. یافته‌ها: 457 دانشجو (8/41% پسر و 2/58% دختر) در 29 رشته تحصیلی در مطالعه شرکت نمودند. میانگین نمره کل و انحراف معیار آگاهی دانشجویان (1/2±56/5)، درک خطر عمومی (12/3±8/21)، درک خطر فردی(2±25/7) و قصد رفتاری دانشجویان (28/3±99/14) می‌باشد. بین میانگین نمرات درک خطر و قصد رفتاری با جنسیت، آگاهی و درک خطر با سن، آگاهی، درک خطر و قصد رفتاری با سطح تحصیلات و رشته تحصیلی دانشجویان رابطه معنی‌داری دیده شد (05/0P<). نتیجه‌گیری: با توجه به در معرض خطر بودن دانشجویان علوم پزشکی و پایین بودن قصد رفتاری آن‌ها در خصوص انجام اقدامات پیشگیرانه، بررسی موانع و فاکتورهای موثر در این خصوص و همچنین انجام مداخلات آموزشی ضروری به نظر می‌رسد.

Title: بهینه سازی فرایند فنتون جهت حذف کل کربن آلی از محیط آبی حاوی آنتی بیوتیک آموکسی سیلین با استفاده از روش آماری تاگوچی

http://hsr.mui.ac.ir/index.php/jhsr/article/view/1316

مقدمه: امروزه آموکسی سیلین یکی از پر مصرف‌ترین آنتی‌بیوتیک‌ها در سراسر دنیا می‌باشد که انتشار آن به محیط زیست اثرات بهداشتی و زیست محیطی متعددی در پی دارد. هدف از این تحقیق بهینه‌سازی فرایند فنتون جهت افزایش میزان معدنی‌سازی آموکسی سیلین موجود در محیط آبی می‌باشد. روش‌ها: در این مطالعه جهت بررسی تاثیر عوامل و تعیین شرایط بهینه حذف TOC از محلول آبی حاوی حاوی آموکسی سیلین توسط فرایند فنتون 5 پارامتر TOC ورودی، غلظت اولیه پراکسید هیدروژن، آهن دوظرفیتی، PH و زمان واکنش هر کدام در 4 سطح مختلف مورد بررسی قرار گرفتند. آزمایشات هرکدام 2 بار تکرار شدند و در نهایت شرایط بهینه و درصد تاثیر هر عامل توسط روش آماری تاگوچی به دست آمد. یافته‌ها: بازده حذف TOC از محیط آبی با غلظت کل کربن آلی ورودی 6، 40، 87 وmg/L 193 به ترتیب برابر 165/37، 285/38، 16/35 و 24/17٪ بود. بهینه‌سازی با روش تاگوچی نشان داد که عوامل مورد نظر در حذف TOC توسط فرایند فنتون، TOC ورودی mg/L 40، H2O2 اولیه mg/L 500، Fe2+ اولیه  mg/L50، PH برابر5/3 و زمان واکنش 10 دقیقه با میزان اهمیت به ترتیب 357/2، 58/73، 837/11، 137/4و 089/8 ٪ بوده‌اند. نتیجه‌گیری: در این مطالعه حداکثر راندمان حذف آموکسی‌سیلین و TOC به ترتیب برابر با 66/99 و 285/38٪ به دست آمد.  نتایج نشان می‌دهد که بر خلاف تجزیه کامل آموکسی سیلین، حذف کامل TOC رخ نداد که این امر می‌تواند به دلیل تولید محصولات جانبی مقاوم به معدنی‌سازی در طی فرایند فنتون باشد. به علاوه از این فرایند می‌توان به عنوان یک واحد پیش تصفیه جهت افزایش میزان معدنی سازی فاضلاب حاوی آموکسی سیلین استفاده نمود.

Title: شیوع بارداری ناخواسته و برخی عوامل مرتیط با آن در بین استفاده‌کنندگان روش منقطع پیشگیری از بارداری در مراکز بهداشتی –درمانی شهرستان گرگان در سال 1389

http://hsr.mui.ac.ir/index.php/jhsr/article/view/845

مقدمه: هر سال تعداد زیادی از زنان در اثر بارداری ناخواسته و سقط غیر ایمن جان خود را دست می دهند. با توجه به اهمیت بارداری ناخواسته مطالعه حاضر با هدف تعیین شیوع بارداری ناخواسته و برخی عوامل مرتبط با آن در بین استفاده‌کنندگان روش منقطع پیشگیری از بارداری در مراکز بهداشتی-درمانی شهرستان گرگان در سال 1389طراحی و اجرا شد.                                                                                                 روش‌ها: در این مطالعه توصیفی-تحلیلی است که در سال 1389 انجام شد.339 نفر از زنان استفاده کننده از روش منقطع پیشگیری از بارداری مراجعه‌کننده به مراکز بهداشتی –درمانی شهرستان گرگان به روش نمونه‌گیری دو مرحله‌ای انتخاب شدند. داده‌ها توسط پرسشنامه‌ای محقق ساخته که روایی آن مورد تایید بود به روش مصاحبه جمع‌آوری شد و با استفاده از نرم‌افزارآماریSSPS نسخه 16 و آزمون‌های کای اسکوئر و دقیق فیشر مورد تجزیه و تحلیل قرار گرفت.                           یافته‌ها: میانگین سنی زنان مورد مطالعه 45/7±10/32بود. 13% زنان بارداری ناخواسته داشتند. بین بارداری ناخواسته با سن، شغل، تحصیلات و تعداد فرزندان زنان مورد مطالعه و همسرانشان ارتباط آماری معنی‌داری مشاهده نگردید. اما با كاهش سن آخرين فرزند بارداري ناخواسته افزايش معناداري را نشان داد.همچنین بین محل سکونت با بارداری ناخواسته ارتباط آماری معنی داری مشاهده شد، به طوری که بارداری ناخواسته در مناطق شهری بیش از مناطق روستایی بود. نتیجه‌گیری: باردار ي ناخواسته در زنان گرگانی نسبتاً بالا است. بنابراین پیشنهاد می‌شود زنان واجد شرایط در رابطه با دلایل بارداری ناخواسته، استفاده از روش‌های پیشگیری از بارداری و عوارض بارداری ناخواسته آموزش داده شوند.

Title: ارزیابی پرتو فرابنفش تابش شده از لامپ‌های فلورسنت فشرده (کم مصرف)

http://hsr.mui.ac.ir/index.php/jhsr/article/view/1134

مقدمه: یکی از روش‌های بهینه‌سازی مصرف انرژی جایگزینی لامپ‌های التهابی با لامپ‌های فلورسنت فشرده می‌باشد در این لامپ‌ها به علت ماهیت تولید روشنایی اشعه فرابنفش تولید و منتشر می‌شود. مطالعه حاضر با هدف ارزیابی پرتو فرابنفش تابش شده از لامپ‌های فلورسنت فشرده انجام گرفت که به طور گسترده در ایران مورد استفاده قرار می‌گیرند. روش‌ها: این مطالعه بر روی 16 لامپ فلورسنت فشرده (4 مارک مختلف) در 8 توان مختلف در آزمایشگاه عوامل فیزیکی دانشکده بهداشت انجام گرفت. اندازه‌گیری پرتو فرابنفش در فاصله 10 سانتی‌متری و در سه طیف UVA،UVB و UVC اندازه‌گیری شد. داده‌ها با استفاده از آزمون آماری ANOVA تجزیه و تحلیل گردیدند. یافته‌ها: شدت پرتو فرابنفش نزدیک یا  Aتابش شده در توان‌های متفاوت در گسترهw/m2 23/0 تا w/m2 06/1 متغیر بود و از نظر آماری دارای اختلاف معنی‌داری بودند (05/.P<) . شدت تابش اشعه فرابنفش متوسط یا B در در گسترهw/m2 0 تا w/m2 02/. در توان‌های متفاوت ( w/m201/0) معنی‌دار نبود (05/.P>). پرتو فرابنفش دور یا C در تمام نقاط اندازه‌گیری شده صفر بود. نتیجه‌گیری: شدت پرتو فرا بنفش در طیف A  و در فاصله  cm10از حباب لامپ در تمام وات‌های اندازه‌گیری شده (به جز لامپ 40 وات) کمتر از حد تماس شغلی 8 ساعته w/m2 041667/1 بود و شدت پرتو فرا بنفش در طیفB  مقادیر اندازه‌گیری شده (به جز لامپ 11 وات) بیشتر از حد مجازw/m2 000003/0بود و شدت پرتو فرا بنفش در طیف C در تمام وات‌ها کمتر از حد آشکارسازی میزان حد تماس شغلی دستگاه اندازه‌گیری بود.

Title: ارتباط‌ سنجي سرمایه اجتماعی درک‌شده و عوامل مرتبط با آن در كاركنان شبكه بهداشت و درمان شهرستان ري تهران

http://hsr.mui.ac.ir/index.php/jhsr/article/view/980

مقدمه: سرمايه اجتماعي به عنوان مجموعه هنجارهاي موجود در سيستم‌هاي اجتماعي موجب ارتقاي سطح همكاري اعضاي جامعه مي‌گردد. عوامل مختلفی بر سطح سرمايه اجتماعي درك شده تاثبر گذار مي‌باشند. بنابراين اين پژوهش با هدف تعیین سرمایه اجتماعی درک‌شده و عوامل مرتبط با آن در كاركنان شبكه بهداشت و درمان شهرستان ري در سال 1391 انجام گردید. روش‌ها: اين پژوهش يك مطالعه مقطعی است كه در سال 1391 در مدت زمان ماه انجام گردید. حجم نمونه 140 نفر و نمونه گيري به روش طبقه بندی بوده است. در این مطالعه ابزار گردآوري اطلاعات شامل اطلاعات دموگرافیک (6 سوال)، و ابزار سرمايه اجتماعي Bullen (36 سؤال) استفاده گردید. داده‌های جمع‌آوری شده با استفاده از نرم افزار SPSS نسخه 18 و آزمون‌های آماري t-test، ANOVA و ضريب همبستگي پيرسون مورد تجزیه و تحلیل قرار گرفت. يافته‌ها: میانگین نمره كل سرمايه اجتماعي شرکت کنندگان در مطالعه 37/14±82/47بود. حیطه‌های سرمايه اجتماعي در افراد مورد مطالعه با متغیرهای سن، جنس، وضعيت تاهل و وضعیت اقتصادی ارتباط معنی‌داری را نشان داد (05/0P<). به علاوه بين حيطه‌هاي سرمايه اجتماعي با سطح تحصيلات، وضعيت استخدامي و محل اشتغال ارتباط آماری معنی‌داری مشاهده نشد (05/0P>).  نتیجه‌گیری: باتوجه به یافته‌های حاصل، تقويت سرمايه اجتماعي در یک جامعه منجر به بهبود فعاليت‌هاي اجتماعي كاركنان خواهد شد بنابراين در برنامه‌ريزي‌هاي مربوط به سلامت كاركنان به توزیع برابر امکانات و تسهيلات رفاهي توجه نمود.

Title: بررسي ارتباط بين حمايت اجتماعي و مذهبي درك شده با وضعيت سلامت زنان و مردان سالمند

http://hsr.mui.ac.ir/index.php/jhsr/article/view/1090

مقدمه: سالمندی دوران حساسی از زندگی است و توجه به مسایل و نیازهای این مرحله یک ضرورت اجتماعی است. دستیابی به سلامت سالمندان و پیشگیری از بروز و ابتلای آنان به بیمارهای مزمن و کاهش شیوع بیماری‌ها در آنان اولین گام شناسایی وضعیت سلامت و عوامل مؤثر بر آن در هرجامعه بوده و یکی از عوامل تاثیرگذار بر این مسأله حمایت اجتماعی ومذهبی می‌باشد. لذا این مطالعه با هدف تعیین تاثیر حمایت اجتماعی و حمایت مذهبی درک شده بر وضعیت سلامت سالمندان انجام شد. روش‌ها : این پژوهش مطالعه توصیفی تحلیلی از نوع مقطعي كه بوده روی 120 نفر از سالمندان 65 سال و بالاتر انجام شد. شیوه نمونه‌گیری به صورت تصادفی ساده از مراکز بهداشتی درمانی شهر سی سخت استان کهکیلویه و بویراحمد بود. ابزار جمع‌آوری داده‌ها پرسشنامه‌ای سه قسمتی بوده که بخش اول مربوط به حمایت اجتماعی درک شده، بخش دوم حمایت مذهبی درک شده و در بخش سوم برای اندازه‌گیری وضعیت سلامت سالمندان از پرسشنامه 12 SF استفاده گردید. روایی و پایایی پرسشنامه در مطالعات قبلی به تایید رسیده است. داده‌ها از طریق مصاحبه ساختارمند (حضوری و تلفني) با سالمندان جمع‌آوری گردیده و با استفاده از نرم‌افزار آماری SPSS نسخه 5/16 و آزمون‌های آماری تی‌مستقل آنالیز واریانس یک‌طرفه و ضریب همبستگی اسپیرمن مورد تجزیه و تحلیل قرار گرفت. سطح معناداری 05/0در نظر گرفته شد. یافته‌ها: نتایج نشان داد ارتباط معنی‌دار آماری بین حمایت مذهبی و حمایت اجتماعی درک شده با وضعیت سلامت سالمندان مورد مطالعه ارتباط معنی‌دار آماری بود (378/1=  r) و (05/0 < P). آزمون تی مستقل نشان داد بین جنس و وضعیت سلامت تفاوت معنادار وجود داشت به گونه‌ای که زنان نسبت به مردان از وضعیت سلامت پایین‌تری برخوردار بودند (05/0 < P). همچنین زنان سالمند در جمعیت مورد مطالعه نسبت به مردان از حمایت مذهبی (05/0< P) و حمایت اجتماعی درک شده (05/0< P) پایین‌تری برخوردار بودند. نتیجه‌گیری: نتایج این مطالعه اهمیت حمایت اجتماعی و مذهبی را در سلامت سالمندان مورد تایید قرار می‌دهد لذا لازم است حمایت مذهبی درک شده و حمایت اجتماعی درک شده به عنوان یک تأمین کننده قوی در وضعیت سلامت در طراحی مداخلات ارتقا سلامت سالمندان مورد توجه قرار گیرد.

Title: ارزیابی گروه های شغلی و توانایی کارکنان با استفاده از شاخص فشار نسبی (RSI) و شاخص توانایی کار(WAI)

http://hsr.mui.ac.ir/index.php/jhsr/article/view/1113

مقدمه: به منظور کاهش حوادث شغلی، بیماری‌های مرتبط با کار و همچنین بهبود رضایت‌مندی از شرایط کاری، انجام ارزیابی‌های شغلی ضروری می‌باشد. هدف از انجام این مطالعه، به کارگیری شاخص‌های ارزیابی ارگونومیک شاخص فشار نسبی (RSI) و شاخص توانایی کار (WAI)، جهت ارزیابی گروه‌های شغلی و توانایی انجام کار کارکنان بود. روش‌‌ها: این مطالعه بر روی 194 نفر مرد شاغل در صنایع نساجی انجام شد. به منظور انجام ارزیابی، از شاخص ماکروارگونومی فشار نسبی (RSI) و شاخص توانایی کار (WAI) استفاده شد. نتایج به دست آمده با استفاده از نرم افزار SPSS نسخه 20 و آزمون‌های آماری رگرسیون خطی چندگانه، t-test و ANOVA مورد تجزیه و تحلیل قرار گرفت. یافته‌ها: نتایج شاخص RSI برای 15 گروه مشاغل نشان داد که تمامی گروه‌ها در منطقه سبز یا ایمن (RSI بزرگتر از6/7) قرار دارند. نتایج حاصل از شاخص WAI هم نشان داد که تنها گروه شغلی اپن دارای توانایی کار خوب بود و بقیه گروه‌های مشاغل دارای توانایی انجام کار متوسط بودند نتیجه‌گیری: ارزیابی ماکروارگونومی مشاغل نشان داد که 15 گروه شغلی برای کارکنان ایمن و بهره‌ور بودند. شغل دولاتاب بیشترین مقدار و شغل فلایر کمترین مقدار این شاخص را داشتند. همچنین وضعیت پوسچر بدن در تمامی 15 گروه مشاغل و وضعیت حواس در گروه مشاغل چله پیچی، اتو کنر، بافندگی و تعمیرات در وضعیت زرد یا هشدار قرار داشتند. نتاج WAIنشان داد که تنها گروه شغلی اوپن دارای توانایی کار خوب بود و بقیه گروه‌های مشاغل دارای توانایی انجام کار متوسط بودند شاخص RSI، علاوه بر بررسی محیط، سطوحی از تناسب افراد برای انجام وظایف را تعیین می کند. اما شاخص WAI، با پایش افراد، مفهومی گسترده تر از تناسب یعنی توانایی انجام کار را تعیین می کند.

Title: اپیدمیولوژی خودکشی در استان بوشهر سال 1388

http://hsr.mui.ac.ir/index.php/jhsr/article/view/1114

مقدمه: خودکشی یکی از مصائب و مشکلات بهداشتی جوامع انسانی می‌باشد که آگاهی از میزان‌ها و اپیدمیولوژی آن در هر منطقه می‌تواند به برنامه‌های کنترل و پیشگیری از آن کمک کند. مطالعه طولی ثبت موارد مرگ سالانه در اجرای این تحقیق به کار گرفته شده است. روش‌ها: در يك مطالعه توصيفي تحليلي در سال 1388، فرمهای از پيش تنظيم شده ثبت موارد خودكشي بر اساس میزان‌های شیوع و کشندگی اختصاصی سنی، جنسی، روش به کار گرفته شده در خودکشی و دیگر متغیرهای دموگرافیک در يك دوره يكساله تکمیل و با استفاده از شاخص خطر نسبي و آزمون‌های همبستگی بررسي شدند. يافته‌ها: شیوع خودکشی در جمعیت زنان استان76/5 و در جمعیت مردان استان88/2 در هر صد هزار نفر برآورد گردید .خطر نسبی مرگ از خودکشی زنان نسبت به مردان در جمعیت مورد بررسی برابر 2 با فاصله اطمینان (6/2-89/1) و اقدام به خودکشی 25/2 با فاصله اطمینان (8/3-03/1)میگردد. در مجموع میزان کشندگی خودکشی‌ها در جامعه مورد مطالعه 6/6 ٪ بود که در دو جنس اختلاف معنی‌داری مشاهده نگردید. در87 ٪ موارد روش به کار گرفته شده، مصرف دارو می‌باشد اما فقط 8٪ از کل مرگ‌ها را به خود اختصاص می‌دهد. دارزدن با 100٪ کشندگی مرگبارترین روش بوده و پس از آن خودسوزی با میزان کشندگی 69 ٪ در رتبه دوم  قرار داشت. روش خودسوزی تنها 6٪ از کل موارد راشامل می‌شد اما 71٪ مرگ‌ها راباعث گردیده است. بالاترین میزان کشندگی در مردان بیکار 3/9 ٪ و سپس در زنان خانه‌دار 2/7٪ مشاهده گردید. نتیجه‌گیری: شیوع خودکشی در زنان استان از متوسط کشوری بالاتر و در مردان استان از متوسط کشوری کمتربود که مشابه مناطق محرومتر کشور میباشد.پرخطرترین گروها برای خودکشی های موفق زنان متاهل خانه دار کم سواد و مردان بیکار با سواد پایین، تشخیص داده شدند و بیشترین خودکشی‌های ناموفق در سنین کمتر از 25 سال بیشتر در زنان باسواد اعم از مجرد و متاهل و سپس پسران باسواد مجرد مشاهده گردید.

Title: بررسی آلودگی صدای کوره بلند در يك صنعت فولاد به منظور کنترل صدا

http://hsr.mui.ac.ir/index.php/jhsr/article/view/1303

مقدمه: در صنعت فولاد کوره‌های با سوخت کک که جهت ذوب سنگ آهن و تولید چدن مورد استفاده قرار می‎گیرند از منابع صدای آزاردهنده محسوب می‎گردند. هدف مطالعه حاضربررسی آلودگی صدای کوره بلند يك صنعت فولاد و مطالعه ویژگی‎های صدای آن به منظور طرح کنترل صدا بود.    روش‌ها: اندازه‎گیری تراز صدا و تجزیه فرکانسی آن با استفاده از ترازسنج صدا مدلCell.450 و دزیمتری صدا با استفاده از دزیمتر مدلTES-1345انجام گردید. توزیع تراز صدا در محوطه کوره بصورت نقشه صوتی با استفاده از نرم افزار SURFERتهیه گردید و ویژگی‎های عایق‎بندی صوتی اتاق کنترل کوره و اتاق استراحت کارگران کوره مورد تحلیل آکوستیکی قرارگرفت. اصلاح اتاق استراحت کارگران و طراحی مجدد درب و پنجره اتاق کنترل پیشنهاد و میزان تاثیر مداخلات برآورد گردید.   یافته‎ها:تراز کلی فشار صوت در محوطه کوره dB(Lin) 3/90 و فرکانس غالب آن Hz4000 در شبکه  خطی (Lin) تعیین شد. میزان افت انتقال صوت دیوار جداکننده اتاق کنترل و اتاق استراحت کارگران کوره به ترتیب dB 3/10 و dB 23/4  تعیین شد. دز صدای دریافتی كارگران240درصد تعیین گردید. میزان کاهش تراز صوت اتاق‌ها با اجرای طرح‌های کنترلی ارائه شده بر مبنای عایق بندی صوت در واحد کوره dB 30 برآورد شده است. نتیجه‎گیری: عامل اصلی آلودگی صدا در اطراف کوره ناشی از جریان آشفته و متلاطم هوا در کانال‌‍‌های هوای ورودی به کوره بود که با توجه به امکان سنجی صورت گرفته پناهگاه صوتی استاندارد در قالب اتاق کنترل و استراحت موثرترین روش کنترل  مواجهه کارگران با صدا ارائه و طراحی گردیدکه براین اساس میزان دز صدای دریافتی کارگران به کمتر از 100 درصد کاهش خواهد یافت.
